# How coach leadership behavior influences athletes’ performance: the chain-mediated role of the coach-athlete relationship and psychological fatigue

**DOI:** 10.3389/fpsyg.2024.1500867

**Published:** 2025-01-22

**Authors:** Rui Liu, Shanshan Wang, Jun Li

**Affiliations:** ^1^Institute of Sports Training, Chengdu Sport University, Chengdu, China; ^2^Physical Education Academy (Gymnastics Academy), Chengdu Sport University, Chengdu, China; ^3^Student Work Department, Tulufan Vocational Technical College, Xinjiang, China

**Keywords:** coach leadership behavior, mediation, influence mechanism, coach-athlete relationship, athlete sport performance

## Abstract

**Objective:**

Athletes’ psychological quality and competitive level are deeply influenced by coaches’ leadership behavior. It is of far-reaching significance to systematically investigate the relationship between them for carrying out scientific training and improving athletes’ competitive level. This study aims to investigate the relationships among coach leadership behavior, the coach-athlete relationship, psychological fatigue, and athletes’ performance, providing insights into enhancing athletes’ sports performance.

**Methods:**

Using simple random sampling, 556 athletes (44.60% female) were recruited from professional training teams in the Xinjiang and Shanxi provinces of China as the study sample. The sample includes 47 s-class national athletes, 276 first-class national athletes, 171 master-class athletes, and 62 international-level athletes. Data were collected through offline surveys using the Coach Leadership Behavior Scale, the Tennis Performance Scale, the Coach-Athlete Relationship Questionnaire, and the Athlete Psychological Fatigue Questionnaire.

**Results:**

Democratic leadership behavior, autocratic leadership behavior, training and instruction behavior, social support behavior, and positive feedback behavior are positively correlated with the “coach-athlete” relationship and athlete performance, and negatively correlated with psychological fatigue. The “coach-athlete” relationship and psychological fatigue can serve as both simple mediators and chain mediators between democratic leadership behavior, autocratic leadership behavior, training and instruction behavior, social support behavior, positive feedback behavior, and athlete performance.

**Conclusion:**

This study systematically explored the complex relationships among coach leadership behavior, the coach-athlete relationship, psychological fatigue, and athletes’ sports performance. The findings suggest that positive coach leadership behavior may contribute to the development of athletes’ performance. Furthermore, the study underscores the significance of the coach-athlete relationship and psychological fatigue as key mechanisms through which coach leadership behavior influences athletes’ sports performance.

## 1 Introduction

As the direct leaders of athletes in competitive sports and the initiators and drivers of leadership behaviors (Gao et al., 2021), coaches’ leadership styles and behaviors have a significant impact on athletes’ performance and skill development (Wang, 2023). The differences in coaching strategies, training methods, coaching philosophy, and planning among coaches are key factors that distinguish various leadership styles, and they form the foundation of a coach’s distinctive leadership style. This unique leadership style shapes the behaviors that motivate athletes to adjust both physically and mentally, fully immerse themselves in competition, and perform at their best. Such leadership behaviors are critical for athletes to achieve their competitive goals as scheduled (Zhang and Ren, 2023) athletes’ sports performance is an important indicator for evaluating an athlete’s competitive level. A systematic study of this topic can help uncover the deep relationships between coaches and athletes, clarify the internal mechanisms affecting athletes’ performance, and ultimately assist coaches in purposefully and effectively organizing their coaching activities. This, in turn, provides a scientific basis for developing athletes’ self-efficacy (Cai and Wu, 2023), physical and mental fitness (Wang, 2023), personality development, and competitive abilities (Chien et al., 2019).

Although numerous studies have systematically explored how to improve athletes’ performance—such as the impact of environmental changes on performance (Shi et al., 2024), the effects of surface material properties on athletes’ sports performance (Wang, 2024), and the influence of external substance intake on the body’s internal environment and performance (Fan et al., 2024), very few have focused on the crucial role coaches play in athletes’ performance development. According to literature reviews, only Wang (2023) has researched the relationship between coach leadership behaviors and athletes’ performance. However, due to the highly complex nature of the coach-athlete relationship, existing studies have not adequately explained the potential mechanisms linking the two. Specifically, current research tends to overlook the significant influence of coach behaviors on athletes’ performance and how coaches, through their leadership behaviors, impact the development of athletes’ performance. This has led to insufficient exploration of the specific mechanisms that may exist between them.

From the perspective of sports practice, both coaches and athletes play vital roles. Under the influence of coaching, athletes exhibit the most noticeable improvements in their competitive performance, while psychological cognition, personality, and social relationships are also affected. Furthermore, as leaders, managers, and strategists of the entire sports team, coaches’ behaviors significantly influence the development of the entire team (Sun and Zhang, 2024). From the perspective of self-determination theory, the behavior of coaches, driven by their subjective agency, is influenced by their psychological needs and affects the psychological fatigue. This, in turn, can have a significant impact on the relationship between the coach and the athlete (Liu et al., 2023). Therefore, this study aims to build upon existing research to deeply explore the internal relationships through which different coaching leadership behaviors affect athlete performance. It focuses specifically on the chain mediation effects of the coach-athlete relationship and psychological fatigue a psychological state caused by sustained high-demand cognitive activity, characterized by feelings of fatigue and lack of energy (Boksem and Tops, 2008), which refers to the interrelated influence among mediating variables, such as X first influencing M1, which then affects M2 and ultimately impacts Y. This approach not only helps further clarify the underlying mechanisms between coaching leadership behaviors and athlete performance but also aids in revealing practical interventions for improving athlete performance. Therefore, the significance of this study lies in enhancing our understanding of how coaching leadership behaviors influence the development of athlete performance and improving coaching effectiveness.

In summary, this study, by focusing on the coach-athlete relationship and psychological fatigue as mediators between coaching leadership behavior and athletes’ sports performance, enriches the theoretical framework of this research area. It provides new evidence for interventions in athletes’ sports performance, offering a more nuanced understanding of the indirect mechanisms influencing athletes’ performance, and ultimately serves as a theoretical reference to help coaches more effectively enhance athletes’ sports performance.

## 2 Literature review and research hypotheses

### 2.1 The direct effect of coach leadership behaviors on athlete performance

Athlete performance, as a direct reflection of an athlete’s competitive level and an influential factor in competition outcomes, has long been a key focus in the fields of sports psychology and training science. Tian (2000) noted in his research that athletes’ sports performance is the comprehensive external manifestation of an athlete’s technical, psychological, physical, tactical, and intellectual abilities during training or competition. It reflects the quality of an athlete’s training or competition level (Liu et al., 2016), and is a critical capability that athletes must continuously develop throughout their career. Coach leadership behavior is a set of behaviors displayed by coaches in influencing the training and competition processes of athletes. It is a key concept in sports psychology that reflects the relationship between coaches and athletes. Specifically, coaches’ leadership behaviors—such as organizational planning, decision-making, communication, and motivation—impact athletes’ psychological cognition, actions, emotional regulation, and sense of belonging (Gao, 2007).

Coach leadership behaviors are typically categorized into democratic leadership behavior, authoritarian leadership behavior, training guiding behavior, social support behavior, and positive feedback behavior. Each type of leadership behavior affects the development of athlete performance in different ways. For instance, democratic leadership behavior encourages athlete participation, enabling them to express opinions during training or competition and improve performance through harmonious interactions with their coach (Gao, 2007). Athletes under democratic leadership behavior tend to exhibit proactive development. In contrast, authoritarian leadership behavior emphasizes the coach’s dominant planning, which may restrict athletes’ autonomy, resulting in uncertain performance outcomes (Cui, 2007). However, authoritarian leadership behavior can also provide strong guidance for athletes with weaker self-discipline. The scientific development of athlete performance under this leadership style remains a matter of debate (Qian et al., 2016). Furthermore, effective leadership plays a crucial role in optimizing team operations (Cotterill, 2013). Training guiding behavior can inspire and internalize motivation in athletes, ultimately enhancing their performance (Cai and Wu, 2013). Social support behavior, which fosters closer relationships between coaches and athletes and enhances athletes’ sense of social belonging (Yan, 2014), can improve performance by addressing athletes’ personal, familial, and self-related needs. Additionally, performance improvement relies on a proper incentive mechanism, known as positive feedback behavior. Research shows that appropriate motivation significantly enhances athlete performance (Perry and Stotsky, 2012).

Based on this analysis, it is evident that all types of leadership behaviors impact athlete performance. Therefore, this study proposes the following hypotheses: H1a: Democratic leadership behavior significantly impacts athlete performance. H1b: Authoritarian leadership behavior significantly impacts athlete performance. H1c: Training guiding behavior significantly impacts athlete performance. H1d: Social support behavior significantly impacts athlete performance. H1e: Positive feedback behavior significantly impacts athlete performance.

### 2.2 The mediating role of the coach-athlete relationship (CAR)

Extensive research indicates a complex mediating mechanism between coach leadership behavior and athlete performance. Competitive sports performance is the result of joint efforts between coaches and athletes. Coach leadership behavior may influence the development of athlete performance through the coach-athlete relationship (CAR). According to interdependence theory, CAR is often seen as an interpersonal exchange between an individual with experience needs and one providing experience. In this relationship, the individual lacking experience gains positive feedback behavior that contributes to their capability development (Jowett and Cockerill, 2003), leading to an unconscious emotional bond (Jowett and Ntoumanis, 2004) and emotional dependency at the cognitive level (Smoll and Smith, 1989), ultimately affecting athlete performance.

However, the outcome is contingent on the quality of the CAR. On one hand, inappropriate leadership behavior that does not align with the athlete’s subjective needs may lead to a rupture in the CAR, negatively impacting performance (Guo and Hu, 2011). On the other hand, proper leadership behavior can harmonize the relationship, fostering high expectations for better performance (Ye et al., 2016a, 2016b) and promoting performance through positive interactions.

One dominant theory regarding CAR is Jowett’s 3C model, which includes closeness, commitment, and complementarity (Yang and Jowett, 2010). Using this model, Jowett found that athlete personality influences CAR and, consequently, athlete performance (Jowett et al., 2012). Similarly, Yang et al. discovered that changes in a coach’s personality—specifically increased conscientiousness and extraversion—positively influence CAR and athlete performance (Yang et al., 2015). Other research has demonstrated that a positive CAR can predict better performance and enhance athletes’ qualities in facing challenges (Chang and Chen, 2023). As an important relationship in competitive sports, CAR is influenced by coach leadership behavior and profoundly impacts athlete performance. Based on this, the following hypotheses are proposed: H2a: CAR significantly mediates the relationship between democratic leadership behavior and athlete performance. H2b: CAR significantly mediates the relationship between authoritarian leadership behavior and athlete performance. H2c: CAR significantly mediates the relationship between training guiding behavior and athlete performance. H2d: CAR significantly mediates the relationship between social support behavior and athlete performance. H2e: CAR significantly mediates the relationship between positive feedback behavior and athlete performance.

### 2.3 The mediating role of psychological fatigue

Psychological fatigue (PF) may be another important internal factor in the relationship between coach leadership behavior and athlete performance. PF is considered a key outcome of coach leadership behavior (Liu et al., 2023). According to self-determination theory, an individual’s participation in activities is influenced by their perceived ability to make choices that meet intrinsic needs, motivational goals, and environmental information, ultimately driving autonomous decision-making (Deci and Ryan, 2000). Research has shown that a coach’s failure to provide appropriate support can trigger PF in athletes (DeFreese and Smith, 2014). Inappropriate leadership behavior can lead to dissatisfaction during training and competition, resulting in negative emotions, PF, psychological harm, and poor performance (Li et al., 2017). PF not only impairs cognitive functioning but also negatively impacts psychological well-being and performance enhancement (Lorist et al., 2005). For example, a study on the effects of PF on swimmers’ brain activity confirmed that PF has detrimental effects on performance and neural activity, suggesting that real-time monitoring of PF could mitigate its impact on performance (Li et al., 2023). However, research has also found that positive coach support can reduce the occurrence of PF (Guo and Yang, 2017). Based on this inference, psychological fatigue is likely to be a mediator in the relationship between coaching leadership behaviors and athletes’ sport performance. However, current research on coaching leadership behaviors and athletes’ sport performance mainly focuses on exploring conditional variables such as psychological preparation (Wang, 2023) and sports anxiety (Yan, 2023), with little attention paid to the role of psychological fatigue as a mediator in the relationship between coaching leadership behaviors and athletes’ sport performance. Therefore, this study proposes the following hypotheses: H3a: psychological fatigue plays a significant mediating role in the relationship between democratic leadership behavior and sport performance; H3b: psychological fatigue plays a significant mediating role in the relationship between authoritarian leadership behavior and sport performance; H3c: psychological fatigue plays a significant mediating role in the relationship between training guiding behavior and sport performance; H3d: psychological fatigue plays a significant mediating role in the relationship between social support behavior and sport performance; H3e: psychological fatigue plays a significant mediating role in the relationship between positive feedback behavior and sport performance.

### 2.4 The chain mediating role of CAR and psychological fatigue

In addition to their individual influences, CAR and PF are important factors that affect athlete performance and are shaped by coach leadership behavior (Gao et al., 2021; Guo and Yang, 2017). Therefore, CAR and PF may play a chain-mediating role in the relationship between coach leadership behavior and athlete performance. CAR, characterized by emotional, cognitive, and behavioral exchanges between coaches and athletes, affects both athletic and psychological development (Jowett, 2009). Research has shown that CAR quality can predict athlete PF, with lower CAR quality increasing PF (Isoard-Gautheur et al., 2015; Cresswell and Eklund, 2007). In this sense, CAR is a key factor influencing PF. Studies suggest that a high-quality CAR negatively predicts PF, meaning that the better the CAR, the lower the athlete’s PF (DeFreese and Smith, 2014). Existing research indicates that coach leadership behavior can directly affect athlete performance or indirectly influence it through CAR or PF. However, there is a lack of evidence on whether CAR and PF jointly play a chain-mediating role in this relationship. Given that performance is crucial for achieving excellent results and is influenced by coach leadership behavior, CAR, and PF, this study proposes the following hypothesis: H4: CAR and PF play a chain-mediating role in the relationship between coach leadership behavior and athlete performance.

## 3 Research methods

### 3.1 Research design and sampling method

Prior to conducting the survey, based on similar studies’ sample effect sizes and considering differences in experimental design, it was determined that at least 300 participants would be necessary to reach the required sample size (Villafaina et al., 2021). Therefore, this study employed a random sampling method, selecting athletes from professional training teams in the provinces of Xinjiang and Shanxi, China. A total of 650 questionnaires were distributed. Due to some questionnaires being incorrectly or incompletely filled out, 556 valid responses were ultimately collected, resulting in an effective response rate of 85.54%. Among the participants, 308 were male (55.40%) and 248 were female (44.60%). The average age of the respondents was 19.34 years (SD = 3.78), with an average training experience of 7.03 years (SD = 3.46). In order to improve the comprehensiveness and authenticity of the study, we invite athletes of all levels as much as possible in the process of selecting athletes, so the final selection of samples involves athletes of different levels, with 47 (8.45%) at the second level, 276 (49.64%) at the first level, 171 (30.76%) at the national level, and 62 (11.15%) at the international level.

The study encompassed a wide range of sports, including basketball, volleyball, martial arts, shooting, triathlon, soccer, boxing, swimming, track and field, table tennis, badminton, tennis, and gymnastics. Although random sampling has certain limitations in statistical analysis, it is advantageous for quickly obtaining relevant information about the target population. Given the constraints of the survey conditions, this method proved to be practical. Direct or indirect contact with the athletes ensured the authenticity of the data collected (see Table 1).

**Table 1:** Demographic Information statistics (*N* = 556).

Characteristics	Frequency (*n*)	Percentage (%)
Gender		
Male	308	55.40
Female	248	44.60
Sports grade		
National secondary level	47	8.45
National level	276	49.64
National athletes	171	30.76
World-class athletes	62	11.15
Education		
High school or below	359	64.57
College or university	134	24.10
Postgraduate	63	11.33
Place of residence		
Urban	119	21.40
Village	437	78.60

### 3.2 Data collection procedure

The survey process for this study was carefully designed. Participants were verbally informed of the research objectives, and informed consent forms were provided to them upon invitation to participate. Consent was obtained from both the professional training team leaders and all athletes involved in the survey. We strictly adhered to the ethical guidelines of the American Psychological Association (APA), providing no rewards to participants while effectively safeguarding their privacy and the confidentiality of their data. This study received approval from the Ethics Review Committee of Chengdu Sport University, which supervised the entire research process (Approval N No. CTYLL2024004). The study was conducted with respect for the participants’ rights.

The data collection and processing were assigned to graduate students specializing in sports psychology, who had undergone professional training to ensure the accuracy of data collection and handling. Before the survey, participants were informed that the data they provided would only be used for research purposes and would not serve any other functions. Strict anonymity was ensured. Detailed instructions for completing the survey were given, and participants were required to independently complete the questionnaire to ensure the authenticity of the research results. To maintain data integrity, the survey was completed and collected on-site.

The on-site completion and collection method was adopted to improve the accuracy of questionnaire responses and ensure high research quality. By adhering to strict data collection and ethical standards, we aimed to conduct a study with high reliability and ethical compliance.

### 3.3 Data analysis

Data processing and analysis were conducted based on the pre-set hypotheses of this study. First, invalid questionnaires were excluded. SPSS 24.0 software, the Bootstrap method, and the SPSS macro Process 4.1 plugin (Model 6) were used for data entry, common method bias testing, descriptive statistical analysis, correlation analysis, and regression analysis. Finally, AMOS 24.0 software was used to construct a structural equation model to verify the relationships between variables, including direct effects, mediation effects, and chain mediation effects.

### 3.4 Measurement tools

#### 3.4.1 Coach leadership behavior scale

This study draws on the “Coaching Leadership Behavior Scale” developed by Qiu (1990), based on the “Leadership Scale for Sports” (LSS) formulated by Chelladurai and Saleh (1980). Compared to other scales for measuring coaching leadership behavior, this scale has more dimensions and a broader scope, making it helpful for a more in-depth examination of the varying degrees of coaching leadership behaviors. It provides stronger support for the development of this study. The scale, which contains 25 items, measures coach leadership behavior from the athlete’s perspective. According to Chelladurai et al.’s classification of coach leadership behaviors, the scale was divided into five dimensions: democratic leadership behavior, authoritarian leadership behavior, training guiding behavior, social support behavior, and positive feedback behavior. Responses were scored on a 5-point Likert scale (1 = strongly disagree, 5 = strongly agree), with higher scores indicating better coach leadership behavior. The model fit indices were as follows: X^2^/df = 1.338, GFI = 0.951, CFI = 0.988, TLI = 0.986, RMSEA = 0.025, SRMR = 0.053, demonstrating good construct validity. The Cronbach’s *α* coefficient for the overall scale was 0.909, with coefficients of 0.892, 0.895, 0.895, 0.853, and 0.884 for the individual leadership behavior dimensions.

#### 3.4.2 Tennis performance scale

This study uses the “Tennis Performance Evaluation Scale” developed by Zhang et al. (2006). The scale includes seven subscales: execution of the plan, loss of composure, low mood, determination, anxiety, flow, and effective tactics, with a total of 28 items. Since some of the respondents participated in non-racket sports, we modified two items in the questionnaire design: “hitting each ball” was changed to “hitting each ball (performing each movement well)” and “worrying about the serve” was changed to “worrying about the serve (worried about not performing the movement well).” This adjustment was made to ensure that athletes from non-racket sports could better understand the questionnaire content and align with the research objectives, thereby enhancing the validity of the scale’s application. The scale uses a 4-point Likert scale ranging from 0 (not at all) to 3 (very strong), with reverse-scored items for numbers 17, 30, and 32. The model fit indices were X^2^/df = 1.815, GFI = 0.927, CFI = 0.974, TLI = 0.972, RMSEA = 0.038, and SRMR = 0.027, indicating good construct validity. The Cronbach’s *α* for the total scale was 0.973, with subscale α values of 0.844, 0.832, 0.827, 0.844, 0.841, 0.843, and 0.845.

#### 3.4.3 Coach-athlete relationship questionnaire

The Coach-Athlete Relationship Questionnaire adapted by Zhong and Wang (2007), based on Jowett and Ntoumanis (2004), was employed. This questionnaire includes 11 items divided into three dimensions: closeness, commitment, and complementarity. Responses were scored on a 5-point Likert scale (1 = strongly disagree, 5 = strongly agree), with higher scores indicating a better coach-athlete relationship. Model fit indices were X^2^/df = 1.544, GFI = 0.979, CFI = 0.994, TLI = 0.992, RMSEA = 0.031, and SRMR = 0.036, indicating good construct validity. The Cronbach’s *α* for the total scale was 0.938, with dimension-specific α values of 0.837, 0.815, and 0.841.

#### 3.4.4 Athlete psychological fatigue questionnaire

The Athlete Psychological Fatigue Questionnaire, adapted by Zhang and Mao (2010) from Raedeke and Smith (2001), was used to measure psychological fatigue. This 15-item questionnaire is divided into three dimensions: reduced sense of achievement, emotional/physical exhaustion, and negative evaluation of sport. Responses were scored on a 5-point Likert scale (1 = strongly disagree, 5 = strongly agree), with higher scores indicating higher levels of psychological fatigue. Model fit indices were X^2^/df = 1.542, GFI = 0.968, CFI = 0.992, TLI = 0.991, RMSEA = 0.031, and SRMR = 0.035, indicating good construct validity. The Cronbach’s α for the overall scale was 0.962, with dimension-specific α values of 0.897, 0.892, and 0.895.

## 4 Results

### 4.1 Common method bias test

Given that the questionnaires were filled out anonymously and involved subjective judgments, there is a potential risk of common method bias. To address this, the Harman single-factor test was conducted through unrotated exploratory factor analysis on 79 variable items. The results showed that eight factors had eigenvalues greater than 1, and the first factor accounted for 32.18% of the variance, which is below the critical threshold of 40%. Therefore, this study concludes that there is no significant common method bias present among the variables, allowing for subsequent analyses.

### 4.2 Descriptive statistics and correlation analysis

Table 2 presents the results of the descriptive statistics and correlation analysis. The findings indicate significant positive correlations between democratic leadership behavior, authoritarian leadership behavior, training guiding behavior, social support behavior, positive feedback behavior, and both athlete performance and the coach-athlete relationship. These behaviors were negatively correlated with athlete psychological fatigue. Additionally, athlete performance was positively correlated with the coach-athlete relationship and negatively correlated with athlete psychological fatigue. The coach-athlete relationship also showed a negative correlation with athlete psychological fatigue.

**Table 2:** Correlation analysis of variables (*N* = 556).

Variable	M	SD	1	2	3	4	5	6	7	8
1 Democratic leadership behavior	3.36	1.14	1							
2 Authoritarian leadership behavior	3.32	1.19	0.354^**^	1						
3 Training guiding behavior	3.29	1.18	0.403^**^	0.316^**^	1					
4 Social support behavior	3.36	1.18	0.330^**^	0.308^**^	0.325^**^	1				
5 Positive feedback behavior	3.43	1.15	0.319^**^	0.284^**^	0.283^**^	0.262^**^	1			
6 Athletes’ performance	1.54	0.83	0.318^**^	0.312^**^	0.319^**^	0.336^**^	0.280^**^	1		
7 “Coach-athlete” relationship	3.36	1.12	0.374^**^	0.306^**^	0.349^**^	0.319^**^	0.266^**^	0.468^**^	1	
8 Psychological fatigue	3.32	1.15	−0.291^**^	−0.322^**^	−0.304^**^	−0.288^**^	−0.236^**^	−0.506^**^	−0.393^**^	1

*p < 0.05, **P < 0.01, and so on.

These significant positive and negative correlations among the variables provide a solid foundation for further research to verify whether the coach-athlete relationship and athlete psychological fatigue mediate the relationship between democratic leadership behavior, authoritarian leadership behavior, training guiding behavior, social support behavior, positive feedback behavior, and athlete performance in a chain mediation model.

### 4.3 Multicollinearity test

Given the significant correlations among the variables, there was a potential risk of multicollinearity affecting the stability of the final results. This study designated athlete performance as the dependent variable, with coach leadership behaviors, the coach-athlete relationship, and athlete psychological fatigue as independent variables for the collinearity diagnosis, standardizing each predictor variable. The results indicated tolerance values of 0.703, 0.738, and 0.771 for the predictor variables, all exceeding 0.1. The corresponding VIF values were 1.423, 1.374, and 1.297, all less than 5. This suggests that there is no multicollinearity issue among the variables, making them suitable for chain mediation effect testing.

### 4.4 Main analysis

#### 4.4.1 Direct effects of variables

Using athlete performance as the dependent variable and coach leadership behaviors (democratic leadership behavior, authoritarian leadership behavior, training guiding behavior, social support behavior, and positive feedback behavior) as independent variables, the results showed that all five dimensions of coach leadership behavior significantly positively influenced athlete performance; Democratic leadership behavior: *β* = 0.631, *p* < 0.01; Authoritarian leadership behavior: *β* = 0.560, *p* < 0.01; Training guiding behavior: *β* = 0.599, *p* < 0.01; Social Support Behavior: *β* = 0.549, *p* < 0.01; Positive Feedback Behavior: *β* = 0.487, *p* < 0.01. This indicates that hypotheses H1a, H1b, H1c, H1d, and H1e are all supported; specifically, higher levels of coach leadership behaviors correlate with improved athlete performance. The reason is that the better the athletes’ own competitive level or athletic ability, the more inclined they are to give higher evaluation when evaluating the coach’s leadership behavior through self-introspection, which shows that the higher the coach’s leadership behavior level, the higher the athletes’ athletic performance level, and the two are in direct proportion.

#### 4.4.2 Mediating effects of the coach-athlete relationship and athlete psychological fatigue

**Figure 1:**
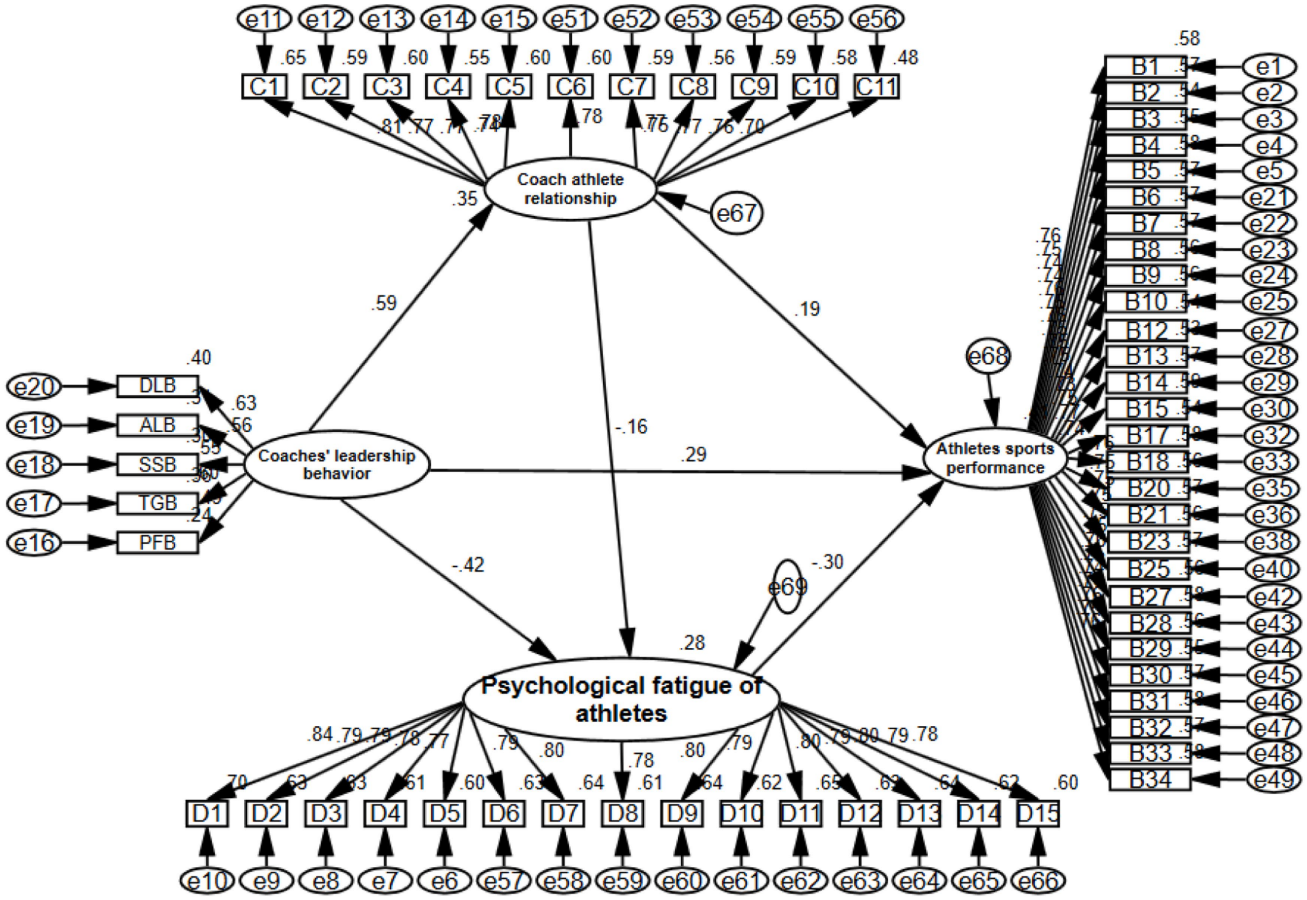
Chain mediation model diagram of the “Coach-Athlete” relationship and psychological fatigue. DLB (Democratic leadership behavior); ALB (Authoritarian leadership behavior); TGB (Training guiding behavior); SSB (Social support behavior); PFB (Positive feedback behavior).

To further verify the research hypotheses (H2a—H2e, H3a—H3e, H4), this study first utilized the Process plugin to test each hypothesis individually. Additionally, to clearly observe the relationships of the mediating variables within the different dimensions of coach leadership behavior and better understand the relationships among mediating variables, AMOS24.0 software was employed to establish a model using a stepwise method (Baron and Kenny, 1986). The results are shown in Table 3 and Figure 1.

**Table 3:** Results of multiple regression analysis.

Suppose	Path	Indirect effect	95%CI	Direct effect	95%CI
H2a	Democratic leadership behavior→ “coach-athlete” Relationship → Athletes’ sports performance	0.0779	[0.0525,0.1068]	0.0770	[0.0232,0.1307]
H3a	Democratic leadership behavior → Psychological fatigue → Athletes’ sports performance	0.0443	[0.0234,0.0657]		
H4a	Democratic leadership behavior→ “coach-athlete” Relationship → Psychological fatigue → Athletes’ sports performance	0.0327	[0.0217,0.0453]		
H2b	Authoritarian leadership behavior→ “coach-athlete” Relationship → Athletes’ sports performance	0.0628	[0.0412,0.0871]	0.0740	[0.0235,0.1244]
H3b	Authoritarian leadership behavior → Psychological fatigue →Athletes’ sports performance	0.0550	[0.0348,0.0766]		
H4b	Authoritarian leadership behavior→ “coach-athlete” Relationship → Psychological fatigue → Athletes’ sports performance	0.0245	[0.0157,0.0349]		
H2c	Training guiding behavior→ “coach-athlete” relationship→ athletes’ sports performance	0.0708	[0.0476,0.0981]	0.0767	[0.0252,0.1282]
H3c	Training guiding behavior → Psychological fatigue → Athletes’ sports performance	0.0481	[0.0279,0.0697]		
H4c	Training guiding behavior→ “coach-athlete” Relationship → Psychological fatigue → Athletes’ sports performance	0.0228	[0.0189,0.0410]		
H2d	Social support behavior→ “coach-athlete” Relationship → Athletes’ sports performance	0.0634	[0.0420,0.0886]	0.1012	[0.0508,0.1516]
H3d	Social support behavior → Psychological fatigue → Athletes’ sports performance	0.0448	[0.0252,0.0659]		
H4d	Social support behavior→ “coach-athlete” Relationship → Psychological fatigue → Athletes’ sports performance	0.0265	[0.0174,0.0385]		
H2e	Positive feedback behavior→ “coach-athlete” Relationship → Athletes’ sports performance	0.0564	[0.0345,0.0812]	0.0837	[0.0329,0.1345]
H3e	Positive feedback behavior → Psychological fatigue → Athletes’ sports performance	0.0369	[0.0172,0.0580]		
H4e	Positive feedback behavior→ “coach-athlete” Relationship → Psychological fatigue → Athletes’ sports performance	0.0247	[0.0154,0.0366]		

The analysis results indicate the following: Firstly, the direct effect of democratic leadership behavior on athletes’ sports performance is 0.0770, with a confidence interval that does not include 0. When the “coach-athlete” relationship and psychological fatigue are separately added, the mediation effects are 0.0779 and 0.0443, respectively, both excluding 0. This suggests that the “coach-athlete” relationship and psychological fatigue play separate and chain mediation roles in the process of democratic leadership behavior influencing athletes’ sports performance. Secondly, the direct effect of authoritarian leadership behavior on athletes’ sports performance is 0.0740, with a confidence interval that does not include 0. When the “coach-athlete” relationship and psychological fatigue are separately added, the mediation effects are 0.0628 and 0.0550, respectively, both excluding 0. This indicates that the “coach-athlete” relationship and psychological fatigue serve as both separate and chain mediators in the influence of authoritarian leadership behavior on athletes’ sports performance. Thirdly, the direct effect of training guidance behavior on athletes’ sports performance is 0.0767, with a confidence interval that does not include 0. When the “coach-athlete” relationship and psychological fatigue are separately added, the mediation effects are 0.0708 and 0.0481, respectively, both excluding 0. This demonstrates that the “coach-athlete” relationship and psychological fatigue have separate and chain mediation effects in the impact of training guidance behavior on athletes’ sports performance. Fourthly, the direct effect of social support behavior on athletes’ sports performance is 0.1012, with a confidence interval that does not include 0. When the “coach-athlete” relationship and psychological fatigue are separately added, the mediation effects are 0.0634 and 0.0448, respectively, both excluding 0. This shows that the “coach-athlete” relationship and psychological fatigue act as separate and chain mediators in the influence of social support behavior on athletes’ sports performance. Fifthly, the direct effect of positive feedback behavior on athletes’ sports performance is 0.0837, with a confidence interval that does not include 0. When the “coach-athlete” relationship and psychological fatigue are separately added, the mediation effects are 0.0564 and 0.0369, respectively, both excluding 0. This indicates that the “coach-athlete” relationship and psychological fatigue play separate and chain mediation roles in the process of positive feedback behavior affecting athletes’ sports performance. This study believes that the reason for the above research results is that athletes are in a relational environment that adapts to their own personality, ability and expectations, and it is easy to have good psychological adaptation at the psychological level, which greatly reduces the probability of psychological fatigue, that is, because athletes with different personalities have different understandings of the “coach-athlete” relationship, especially with the improvement of competitive level, the individual’s dependence on the relational environment is further deepened. It makes the sports practice carried out under the leadership behavior of coaches in different dimensions have a homogeneous influence on the relationship between coaches and athletes and the formation of psychological fatigue, and also makes the athletes’ sports performance develop synchronously.

## 5 Discussion

### 5.1 The impact of coach leadership behavior on athletes’ sports performance

The research findings support hypotheses H1a-H1e, indicating that the five dimensions of coach leadership behavior (Democratic leadership behavior, Authoritarian leadership behavior, Training guiding behavior, Social support behavior, and Positive feedback behavior) all have significant positive impacts on athletes’ sports performance, consistent with previous research results (Wang, 2023). Related research suggests that democratic leadership behavior is more helpful for athletes to overcome burnout (Yu et al., 2024) and stimulate their potential athletes’ sports performance through profound introspection. This introspection stems from coaches leading athletes through encouragement and rewards under the influence of democratic leadership behavior (Smith et al., 2007), which has a significant impact on athletes’ individual psychology and cognition, especially through the “coach-athlete” relationship (Zhu et al., 2017), exerting a notable indirect influence that fosters athletes’ psychological admiration for coaches and enhances athletes’ sports performance through self-reinforced skill levels (Guo et al., 2021). Authoritarian leadership behavior emphasizes athletes’ development of athletic levels under the guidance of coaches’ predetermined training plans, with athletes’ sports performance closely linked to the rationality of these plans (Wang, 2023). Additionally, research points out that long-term stable and scientific training guidance behavior is the most direct and highly relevant factor for improving athletes’ engagement in sports (Cai and Wu, 2013) and achieving excellent results through high-level athletes’ sports performance. This leadership behavior can have an incentive and internalizing effect on athletes (Zhan, 2016). Furthermore, research on coaches’ social support behavior indicates that coaches create an incentive atmosphere that positively promotes athletes’ psychological, emotional, and behavioral aspects, thereby enhancing their athletes’ sports performance (Cai, 2016). In this incentive atmosphere, coaches’ positive feedback behavior is crucial for guiding the development of athletes’ sports performance, better helping athletes psychologically engage in training and competitions (Cai, 2016) to fully leverage their athletic abilities. Even athletes with stronger abilities to receive positive feedback behavior from coaches can, to some extent, exceed their usual levels of athletes’ sports performance. Therefore, coaches should properly handle the relationship between leaders’ behaviors in the process of coaching, and help improve sports performance scientifically. In summary, coach leadership behavior is a significant factor influencing athletes’ sports performance, and there is a positive predictive relationship between each dimension of coach behavior and athletes’ sports performance. Therefore, hypotheses H1a-H1e are supported.

### 5.2 The mediating role of the “coach-athlete” relationship

The research findings support hypotheses H2a-H2e, indicating that the “coach-athlete” relationship has a significant positive impact between the five sub-dimensions of coach leadership behavior and athletes’ sports performance. This suggests that a higher level of coach leadership behavior leads to a better “coach-athlete” relationship and, consequently, better athletes’ sports performance. Conversely, poorer leadership results in a worse relationship and performance. These findings align with previous research (Cao and Cao, 2021; Jin et al., 2022). Meanwhile, studies have confirmed that athletes’ high levels of engagement in sports are due to the positive influence of coach leadership behavior, mediated by a good “coach-athlete” relationship (Liu, 2014). The study also points out that coaches’ behaviors in guiding athletes’ training and providing necessary social support behavior for their athletic development can promote athletes’ engagement in sports. The impacts of democratic, authoritarian, and positive feedback behaviors on athletic engagement need to be realized through the “coach-athlete” relationship. In fact, research results from Yang (2007), Ban and Chen (2013), and Nicholls and Perry (2016) demonstrate a significant positive correlation between coach leadership behavior and the “coach-athlete” relationship, meaning that better coach leadership leads to a better “coach-athlete” relationship, ultimately affecting the development of athletes’ sports performance. The aforementioned research indicates that democratic leadership behavior is conducive to improving the quality and enhancing the quality of the “coach-athlete” relationship. Coach leadership can positively predict the three dimensions of the “coach-athlete” relationship, benefiting the development of athletes’ sports performance. However, it also points out that an unreasonable “coach-athlete” relationship is not only an indirect manifestation of poor leadership behavior but also an obstacle to the development of athletes’ sports performance. If some research points out that under the influence of good relationship, athletes are more willing to express their personal demands, form a strong sense of goal commitment, and are willing to work hard to achieve more ideal competitive performance (Ye et al., 2016a,b). With the positive development of the “coach-athlete” relationship, a harmonious cooperative environment forms between the two main entities in sports practice, effectively gathering positive factors that can enhance athletes’ sports performance, such as psychological positive guidance and training resource investment (Lefebvre et al., 2021), thereby promoting the development of athletes’ sports performance. Therefore, in the future training practice, relevant departments should attach importance to the cultivation of “coach-athlete” relationship and adopt various ways to promote the good development of this relationship, specifically, through gratitude and forgiveness, interpersonal management and conflict mediation. In summary, each sub-dimension of coach leadership behavior can influence athletes’ sports performance through the “coach-athlete” relationship, meaning that there is a positive predictive relationship between the five dimensions of coach leadership behavior and athletes’ sports performance, mediated by the “coach-athlete” relationship. Therefore, hypotheses H2a-H2e are supported.

### 5.3 The mediating role of psychological fatigue

The research findings support hypotheses H3a-H3e, indicating that psychological fatigue has a significant negative impact between the five sub-dimensions of coaches’ leadership behaviors and athletes’ performance. This suggests that higher levels of coaches’ leadership behaviors are associated with lower psychological fatigue and better athletes’ sports performance, and vice versa. These results align with the findings of Zhang et al. (2023), Sarason et al. (1990), and Raedeke and Smith (2004), demonstrating that different dimensions of coaches’ leadership significantly negatively predict athletes’ psychological fatigue. Altahayneh (2003) and Guo et al. (2021) argue that coaches’ leadership behaviors are crucial factors influencing athletes’ psychological fatigue. Furthermore, different dimensions of coaches’ leadership behaviors serve as reliable predictors of psychological fatigue. Good coaches’ leadership behaviors are often associated with better psychological positivity, which can reduce athletes’ psychological fatigue. Conversely, high psychological maladjustment is linked to poor leadership behaviors, leading to increased feelings of psychological fatigue and subsequently affecting athletes’ sports performance development. Additionally, athletes are more likely to be influenced by interpersonal and linguistic environments, where negative messages increase their psychological burden and heighten feelings of psychological fatigue (2006). The study also found that among high-level athletes, irregular training organization, inhumane personnel management, inability to communicate with coaches, and insufficient psychological support are significant factors contributing to increased psychological fatigue. This is attributed to coaches’ authoritarian leadership behaviors. Conversely, athletes with low psychological fatigue often achieve success under the guidance of positive leadership behaviors, which in turn enhances their athletes’ sports performance. These studies suggest a close correlation between psychological fatigue and coaches’ leadership behaviors, with psychological fatigue being a key factor in negatively predicting coaches’ leadership behaviors (Levy et al., 2009; Curtin, 2020; Cahyono et al., 2023). The research also shows that psychological fatigue is the key factor to reduce sports performance. Therefore, in the future practice, athletes themselves can reduce psychological fatigue through self-suggestion, while coaches can reduce athletes’ psychological fatigue by guiding athletes’ attention shift, or chatting and mediation, thus laying the foundation for improving athletes’ sports performance. Athletes with high levels of psychological fatigue cannot affirm their competitive abilities, thereby decreasing their performance, while those with low levels of psychological fatigue have a stronger desire for athletes’ sports performance. Therefore, athletes’ psychological fatigue can effectively reflect their level of athletes’ sports performance (Russell et al., 2019; Pageaux and Lepers, 2018; Habay et al., 2021). Other studies have found that different dimensions of coaches’ leadership behaviors can indirectly affect athletes’ sports performance through psychological fatigue. Zhang and Price agree that coaches’ leadership behaviors are effective predictors of psychological fatigue and, in turn, have a negative impact on athletes’ sports performance (Zhang et al., 2023; Price and Weiss, 2000). Meanwhile, research confirms that an increasing number of athletes experience elevated psychological fatigue due to coaches’ improper leadership behaviors, prompting athletes to seek positive improvement methods to alleviate their psychological fatigue and enhance their athletes’ sports performance (Barcza-Renner et al., 2016). In summary, the sub-dimensions of coaches’ leadership behaviors can influence athletes’ performance through psychological fatigue, indicating a negative predictive role of psychological fatigue between the five dimensions of coaches’ leadership behaviors and athletes’ performance. Therefore, hypotheses H3a-H3e are supported.

### 5.4 The chain-mediating role of the “coach-athlete” relationship and psychological fatigue in the relationship between coaches’ leadership behaviors and athletes’ sports performance

The research findings support hypothesis H4, indicating that the “coach-athlete” relationship and psychological fatigue have a significant chain-mediating effect between coaches’ leadership behaviors and athletes’ performance. This suggests that under the guidance of good coaches’ leadership behaviors, it is more likely to establish a positive “coach-athlete” relationship, effectively reduce psychological fatigue, and subsequently improve athletes’ performance. The reverse is also true. As previous studies have confirmed, the “coach-athlete” relationship and psychological fatigue are mediators in the relationship between coaching leadership behavior and athlete performance. Moreover, existing research has shown a correlation between the “coach-athlete” relationship and psychological fatigue, indicating that better coach-athlete relationships negatively impact athletes’ psychological fatigue, while poor coach-athlete relationships increase psychological fatigue (McGee and DeFreese, 2019; Davis et al., 2018). These findings, which align with the results of this study, further confirm the correctness of the research hypothesis. Additionally, previous research has also confirmed the correlation between the “coach-athlete” relationship and psychological fatigue, showing that better relationships reduce psychological fatigue, while poor relationships increase it. Furthermore, studies have found that improving the coach-athlete relationship not only reduces psychological fatigue but also enhances athletic performance. This highlights the fact that effective coaching leadership behavior can promote a harmonious coach-athlete relationship, leading to a reduction in psychological fatigue and, in turn, improving performance. Therefore, coaching leadership behavior can influence athlete performance through the chain mediation effect of the “coach-athlete” relationship and psychological fatigue, thus supporting the validity of hypothesis H4.

## 6 Conclusions and practical implications

This study demonstrates that coaching leadership behaviors, encompassing democratic leadership behavior, authoritarian leadership behavior, training guiding behavior, social support behavior, and positive feedback behavior, are positively correlated with the “coach-athlete” relationship and athletes’ sport performance, while negatively correlated with psychological fatigue. The research indicates that coaches with better leadership behaviors are more likely to facilitate athletes’ sport performance by establishing positive “coach-athlete” relationships and simultaneously reduce athletes’ psychological fatigue. Notably, this study finds that both the “coach-athlete” relationship and psychological fatigue can independently or sequentially mediate the relationship between coaching leadership behaviors and athletes’ sport performance.

This research carries several practical implications: First, it emphasizes the importance of coaching leadership behavior in the development of athlete performance, providing psychological strategies for athlete development in different coaching leadership behaviors or coaching contexts. Second, it offers insights into coaches’ individual psychological mechanisms, furnishing methods and ideas for evaluating coaches and even the development of sports teams. Third, the findings of this study also have implications for the development of sports practice, aiding in identifying and addressing declines in sport performance caused by the “coach-athlete” relationship and individual athletes’ psychological fatigue. Fourth, the results suggest that the standardized use of coaching leadership behaviors, coupled with the harmonious development of the “coach-athlete” relationship, may help reduce psychological fatigue, enhance sport performance, and achieve more efficient competitive performance.

This study expands the theoretical framework of the relationship between coach’s leadership behavior and athletes’ sports performance, and shows that coach’s leadership behavior not only directly affects sports performance, but also indirectly affects through coach-athlete relationship and psychological fatigue. This discovery is of great significance to psychology and competitive sports, and enriches the understanding of how coaches’ leadership behavior affects athletes’ sports performance. Future research can explore other potential intermediary factors, such as self-efficacy and motivation, to further clarify the relationship mechanism between coaches’ leadership behavior and athletes’ sports performance. In addition, cross-regional research can help to understand how different cultural differences affect this relationship, thus ensuring verification in different regions. With the development of AI, future research can use these technologies to provide more accurate strategies to enhance athletes’ performance.

## 7 Limitations and future directions

Although this study contributes to the exploration of coaching theory and practice, it does have some limitations. First, in terms of the sampling method, the lack of random sampling may limit the generalizability of the study’s findings. Additionally, due to geographical limitations, the study was restricted to only two provinces, and did not take into account potential external influences such as differences in sports habits, culture, or economics. As a result, the study’s findings may not have fully considered the impact of these external factors. Second, this study only used a cross-sectional design, which lacks strong evidence for causal inferences and does not account for potential biases inherent in cross-sectional research. Third, as the data collection primarily relies on self-reported measures, there may be subjective biases that reduce the evidential support for the study’s results.

To address these limitations, first, our next step in research will focus on collecting samples based on various demographic characteristics to enhance the generalizability of the findings. Given the numerous provinces in China and the significant differences between the north and south, east and west, there may be potential factors influencing the research process. Therefore, our future research will emphasize considering these aspects and conducting deep and detailed investigations. Second, in our future studies, we will adopt more objective sample measurement standards to enhance the scientific level of sample collection and processing, For example, with the development of science and technology, we can use new technology to collect and measure objective performance or data obtained through observation, thus further increasing the depth of research. Third, from the perspective of long-term sports development, time may be an important factor influencing the improvement of athletic performance. Therefore, future research will use longitudinal surveys to collect samples and examine the impact of coaching leadership behaviors on athlete performance during the process of change. This will make causal inferences more convincing.

## Data Availability

The raw data supporting the conclusions of this article will be made available by the authors, without undue reservation.
